# Enhancing immunogenicity of novel multistage subunit vaccine of *Mycobacterium tuberculosis* using PLGA:DDA hybrid nanoparticles and MPLA: Subcutaneous administration

**DOI:** 10.22038/ijbms.2019.33962.8079

**Published:** 2019-08

**Authors:** Farzad Khademi, Arshid Yousefi, Mohammad Derakhshan, Adel Najafi, Mohsen Tafaghodi

**Affiliations:** 1 Department of Microbiology, School of Medicine, Ardabil University of Medical Sciences, Ardabil, Iran; 2 Department of Medical Bacteriology and Virology, Qaem University Hospital, School of Medicine, Mashhad University of Medical Sciences, Mashhad, Iran; 3 Nanotechnology Research Center, Pharmaceutical Technology Institute, Mashhad University of Medical Sciences, Mashhad, Iran; 4 Department of Pharmaceutics, School of Pharmacy, Mashhad University of Medical Sciences, Mashhad, Iran

**Keywords:** Immunization, Mycobacterium tuberculosis, Multistage subunit vaccine, MPLA, PLGA:DDA nanoparticle

## Abstract

**Objective(s):**

A new strategy in recent studies is using effective tuberculosis (TB) subunit vaccines combined with appropriate carriers and adjuvants which have shown promising results in preclinical and clinical studies. The aim of the present study was to evaluate the PLGA:DDA hybrid nanoparticles (NPs) for subcutaneous delivery of a novel multistage subunit vaccine along with MPLA adjuvant against *Mycobacterium tuberculosis* (*M. tuberculosis*).

**Materials and Methods:**

PLGA and PLGA:DDA NPs containing HspX/EsxS fusion protein and MPLA were prepared by double emulsion method (w/o/w). After characterization, these NPs were subcutaneously administered to BALB/c mice aged 6-8 weeks old. Immunogenicity of formulations were assessed by measuring the level of IFN-γ, IL-4, IL-17 and TGF-β cytokines as well as IgG1, IgG2a and IgA antibodies using ELISA.

**Results:**

Both particles had spherical shape and smooth surface with 316.7±12.7 nm in size, surface charge of -33±1.7 mV, and encapsulation efficiency of 92.2±2% for PLGA NPs and 249.7±16.7 nm in size, surface charge of 39±1.8 mV, and encapsulation efficiency of 35.7±1.4% for PLGA:DDA NPs. The highest IFN-γ response and also IgG2a and IgG1 antibodies titers were observed in groups immunized with PLGA:DDA/HspX/EsxS/MPLA and PLGA:DDA/HspX/EsxS/MPLA as booster as well as PLGA:DDA/HspX/EsxS and PLGA:DDA/HspX/EsxS as booster.

**Conclusion:**

With regard to effective induction of IFN-γ and IgG2a immune responses, PLGA:DDA hybrid NP along with MPLA adjuvant have good potentials for improving the immunogenicity of HspX/EsxS multistage subunit vaccine as well as promoting BCG efficacy as a BCG prime-boost.

## Introduction

After HIV-1 infection, tuberculosis (TB) is the second fatal infectious disease and its morbidity and mortality in the world was about 1.8 million die in 2015, 1.1 million men, 0.5 million women and 0.2 million children (1-3). It is estimated that nearly one-third of the world’s population are latently infected with TB infection with the possibility of 5-10% of TB reactivation to active form (4, 5). From a century ago, vaccination with BCG, *Mycobacterium bovis* bacilli Calmette-Guérin, is only approved approach to control *Mycobacterium tuberculosis (M. tuberculosis)* (1-3). However, BCG vaccine is not able to: 1) prevent from reactivation of dormant *M. tuberculosis*, 2) induce sufficient immune responses to dormancy expressed antigens compared with secretory antigens expressed by rapid growing bacteria and 3) produce a variable protective efficacy in adolescents and adults (0-80% protection) (2, 6-8). Therefore, using of early- and late-stage TB antigens, as multistage subunit vaccines, to develop an effective vaccine against both acute and stationary stages of infection is highly necessary (7). Multistage subunit vaccines could be administered as a BCG prime-boost before and after TB infection and can promote protective immunity and improve BCG efficacy in terms of long-term immunological memory in adolescence or adulthood as well as in the latent stage of disease (9, 10). Therefore, a 16-kDa heat shock protein of *M. tuberculosis* (HspX protein), expressed by non-replicating bacteria in latent-phase, as well as a 10-kDa ESAT-6 like protein EsxS, expressed by replicating bacteria in early-phase, were two potentially immunodominant antigens recruited for designing a multistage subunit vaccine due to their ability in enhancing both cell-mediated and humoral immune responses in recent studies (11, 12). However, a major issue associated with multistage subunit vaccines is its low immunogenicity. Therefore, it can be helpful to use effective adjuvants or different delivery systems to stimulate the strong immune responses (13, 14). A suitable adjuvant used in TB vaccines should be able to efﬁciently induce Th1 type immune responses (15). To achieve this aim, MPLA (monophosphoryl lipid A) adjuvant which is a detoxiﬁed derivative of lipid A from lipopolysaccharide (LPS) of *Salmonella minnesota* R595 strain can be effective according to previous studies (16). This immunostimulant is a potent activator of toll-like receptor 4 (TLR-4) with negligible TLR-2 activity (16). But, in spite of the positive potentials of adjuvants as strong inducer of innate immune responses as well as stimulant of the transition of responses from innate to adaptive immunity and also from Th2 to Th1, their use have some limitations such as local and systemic side effects (15, 17). However, the ability of nanoparticles (NPs) has been proved as delivery systems for subunit vaccines through: 1) compensate of low immunogenicity of antigens, 2) protection of encapsulated antigen from *in* *vivo* degradation and 3) act as an antigen reservoirs for antigen-presenting cells (APCs) (16, 18). Studies showed that PLGA (poly (lactide-co-glycolide) NPs have several positive potentials including biocompatibility and biodegradability, FDA approval, safety, enhanced colloidal stability and efficient uptake by APCs (19-24). However, compared with positively charged NPs, negatively charged PLGA shows poor stability, weak interaction and cellular uptake and rapid release properties (20, 25). Therefore, using cationic lipids such as DDA, dimethyl dioctadecylammonium bromide, can lead to improve characteristics of the PLGA matrix (20). For this purpose, we used from DDA cationic liposome-forming lipid to develop a novel nano-based vaccine against TB infection. A quaternary ammonium compounds which previously have been reported as an effective adjuvant to stimulate humoral and cellular immune responses. Due to the negative charge on the cell membrane, positively charged polymers such as PLGA:DDA can better connect to the cell membrane. Also it has been demonstrated that liposomes are able to enhance the ability of the vaccines to absorb by the APCs (26-28). The goal of this study was to generate a novel multistage subunit vaccine, HspX/EsxS, combined with MPLA adjuvant as well as encapsulating them in a PLGA:DDA hybrid NP in order to develop an integrated vaccine regimen and investigate the humoral- and cell-mediated immune responses after subcutaneous administration in mice model. These nano-vaccines were also tested as booster for BCG vaccine.

## Materials and Methods

***Materials***

PLGA, poly (D, L-lactide-co-glycolide) (Resomer RG 752 H, lactide:glycolide ratio 75:25) (MW: 4,000-15,000 Da), PVA, (poly (vinyl alcohol) (MW: 89000 to 98000 Da, 99% hydrolyzed), DDA, MPLA and dichloromethane, at analytical grade, were purchased from Sigma-Aldrich (Germany). ELISA assay kit for mouse IFN-γ, IL-4, IL-17 and TGF-β cytokines were purchased from eBioscience (USA). Goat anti-mouse IgG1 and IgG2a secondary antibodies, HRP conjugate were obtained from Invitrogen (USA).

***Animals***

Forty-five specific pathogen-free BALB/c mice aged 6-8 weeks old were obtained from Pasteur Institute (Tehran, Iran) and maintained in cages in animal house of Bu-Ali Research Center. Animals had free access to standard pellet diet (Khorassan Javane Co, Mashhad, Iran) and water throughout the study. Mashhad University of Medical Sciences approved the research protocol and all animal experiments were performed in accordance with the guidelines of Ethical Committee Acts.

***HspX/EsxS fusion protein***

Cloning, expression and purification of HspX/EsxS fusion protein was done as previously described (11). Brieﬂy, initially pGH vector containing *hspX/esxS* gene construct was transformed into *Escherichia coli* strain TOP10 and purified. Then both pGH/*hspX/esxS* and pET-21b (+) plasmids were extracted and digested with the same *HindIII *and *XhoI* enzymes and the desired fragment was ligated into the digested pET-21b (+) plasmid. Colony-PCR and sequencing methods were used to confirm the accuracy of cloning. At the next step, the pET-21b (+)/*hspX/esxS* expression vector was transferred into *E. coli* strain BL21 and protein expression was evaluated by sodium dodecyl sulfate-polyacrylamide gel electrophoresis (SDS-PAGE). Eventually, fusion protein expression was purified by Ni-IDA column and dialysis performance and verified by Western blotting methods.

**Table 1:** Physical characteristics of PLGA and PLGA:DDA hybrid NPs

**Formulation**	**Z-average (nm)**	**Zeta-potential (mV)**	**PDI**	**Encapsulation rate** **(%)**	**Yield** **(%)**
PLGA:DDA	249.7±16.7	39±1.8	0.233±0.07	35.7±1.4	41±2
PLGA	316.7±12.7	-33±1.7	0.218±0.03	92.2±2	50.1±2.1

All data presented as means ± SD (n=3)

**Figure 1:**
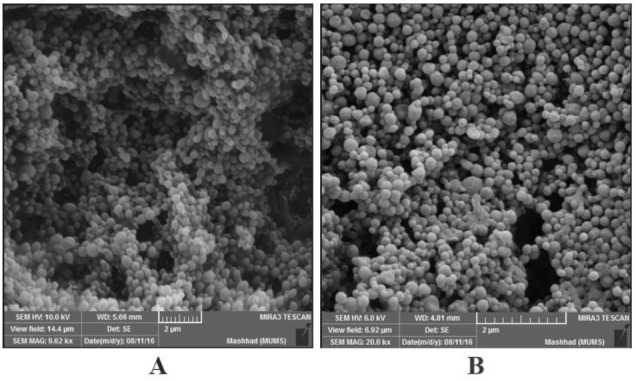
SEM image of PLGA and PLGA:DDA hybrid NPs. A) PLGA NPs containing HspX/EsxS fusion protein. B) PLGA:DDA hybrid NPs containing HspX/EsxS fusion protein and MPLA

PLGA: poly lactide-co-glycolide; DDA: dimethyl dioctadecylammonium bromide

**Figure 2:**
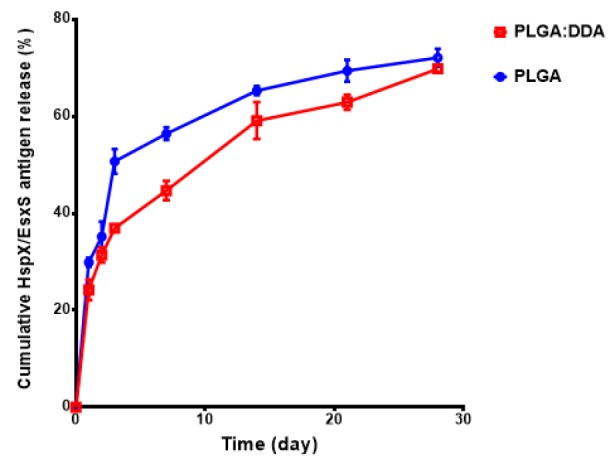
Cumulative release of HspX/EsxS fusion protein from PLGA and PLGA:DDA hybrid NPs. All data presented as means±SD (n=3)

PLGA: poly lactide-co-glycolide; DDA: dimethyl dioctadecylammonium bromide

**Figure 3:**
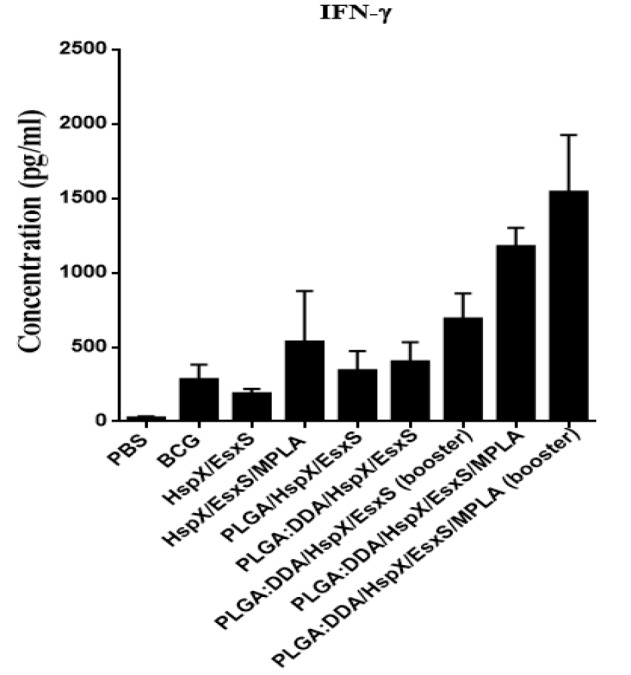
IFN-γ secretion from cultured spleen cells of immunized mice. Subcutaneous immunization of mice were done with different formulations three times at two weeks intervals. Three weeks after ﬁnal vaccination, IFN-γ release in stimulated mice splenocytes with HspX/EsxS and PHA were assessed by an ELISA method. PBS and BCG groups were used as controls. All data presented as means±SEM (n=5)

**Figure 4:**
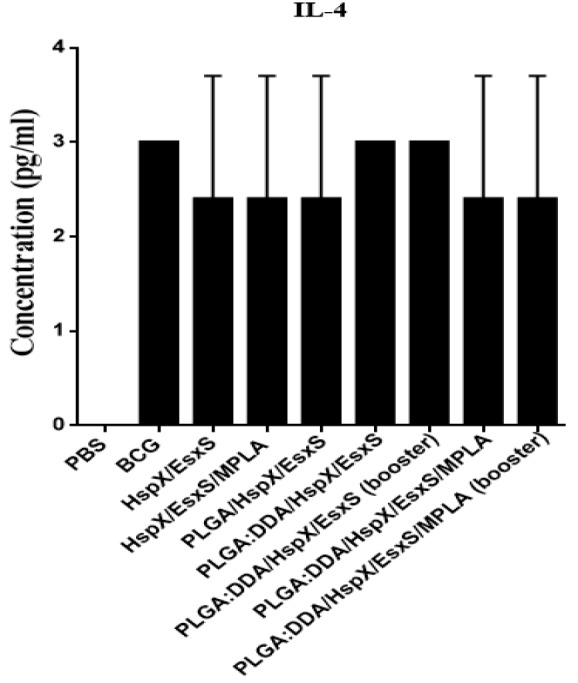
IL-4 secretion from cultured spleen cells of immunized mice. Subcutaneous immunization of mice were done with different formulations three times at two weeks intervals. Three weeks after ﬁnal vaccination, IL-4 release in stimulated mice splenocytes with HspX/EsxS and PHA were assessed by an ELISA method. PBS and BCG groups were used as controls. All data presented as means±SEM (n=5)

**Figure 5:**
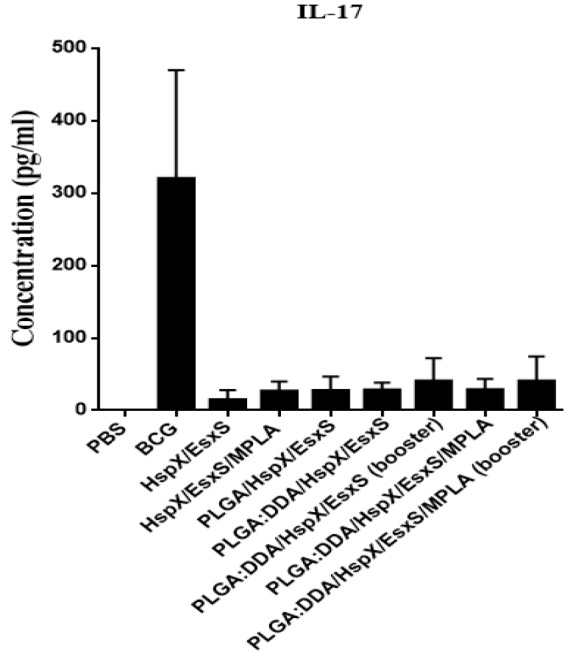
IL-17 secretion from cultured spleen cells of immunized mice. Subcutaneous immunization of mice were done with different formulations three times at two weeks intervals. Three weeks after ﬁnal vaccination, IL-17 release in stimulated mice splenocytes with HspX/EsxS and PHA were assessed by an ELISA method. PBS and BCG groups were used as controls. All data presented as means±SEM (n=5)

**Figure 6:**
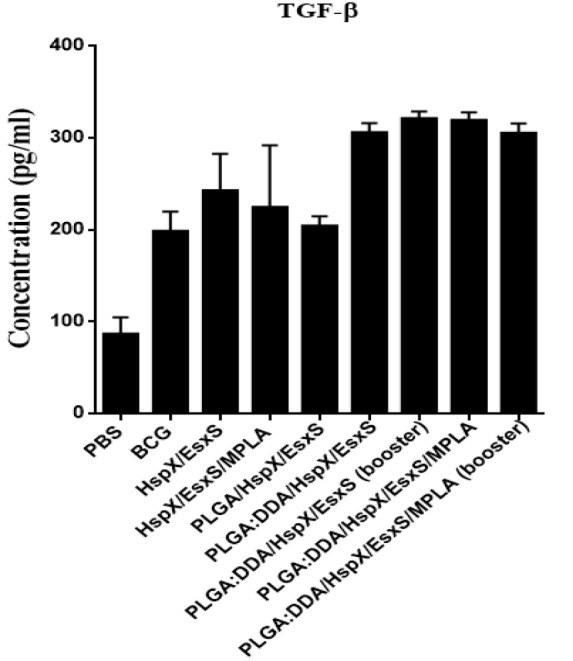
TGF-β secretion from cultured spleen cells of immunized mice. Subcutaneous immunization of mice were done with different formulations three times at two weeks intervals. Three weeks after ﬁnal vaccination, TGF-β release in stimulated mice splenocytes with HspX/EsxS and PHA were assessed by an ELISA method. PBS and BCG groups were used as controls. All data presented as means±SEM (n=5)

**Figure 7:**
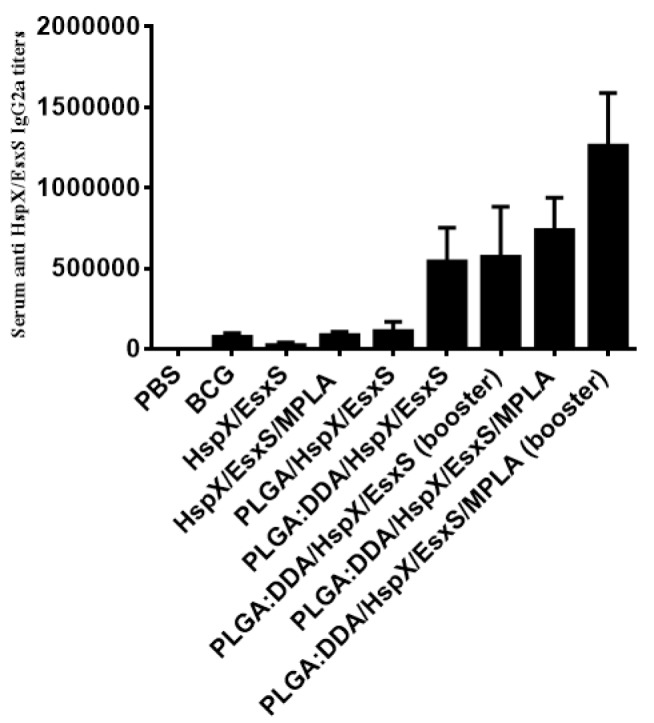
Serum anti-HspX/EsxS IgG2a titers of immunized mice. Subcutaneous immunization of mice were done with different formulations three times at two weeks intervals. Three weeks after ﬁnal vaccination, mice serum sample were obtained and antibody titers were evaluated by an ELISA method. All data presented as means±SEM (n=5)

**Figure 8:**
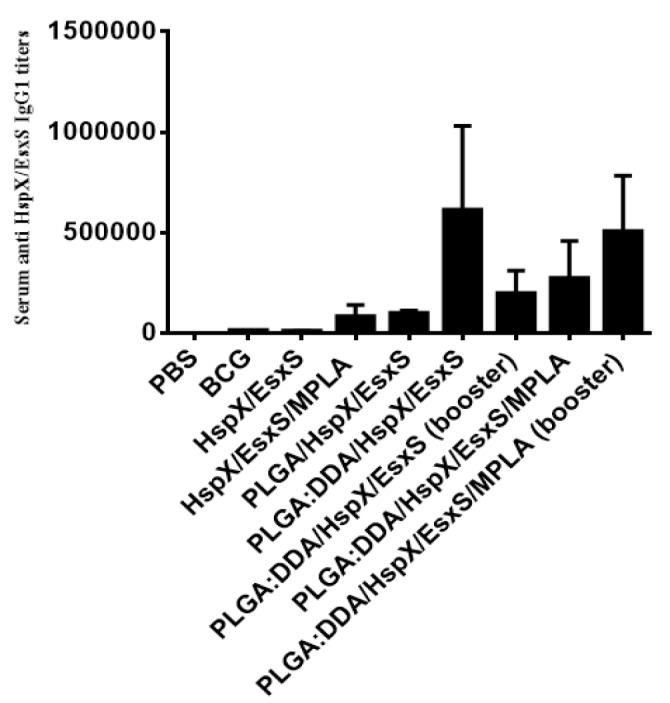
Serum anti-HspX/EsxS IgG1 titers of immunized mice. Subcutaneous immunization of mice were done with different formulations three times at two weeks intervals. Three weeks after ﬁnal vaccination, mice serum sample were obtained and antibody titers were evaluated by an ELISA method. All data presented as means±SEM (n=5)

***Preparation of PLGA:DDA hybrid NPs***

We previously optimized and produced PLGA:DDA hybrid NPs containing HspX/EsxS fusion protein and the adjuvant MPLA by the double emulsion method (w/o/w) (unpublished data). Brieﬂy, 50 mg of PLGA and 10 mg of DDA (60 mg/ml) were weighted and mixed with 10 μl of MPLA solution (5 mg/ml) in 600 µl dichloromethane. By adding 120 μl of an aqueous phase with 1 mg/ml of HspX/EsxS fusion protein into PLGA/DDA/MPLA organic phase and sonication for 30 sec, water-in-oil (w_1_-o) primary emulsion was formed. To establish water-in-oil-in-water (w_1_-o-w_2_) emulsion, primary emulsion was added to 4 ml of 0.5% (w/v) PVA solution and sonicated for 60 sec. The emulsion was added to 20 ml of 0.3% (w/v) of PVA solution and stirred for 24 hr and subsequently centrifuged (18000 *g*, 4 ^°^C for 12 min), purified (three times with 20 ml of ultrapure water) and freeze-dried for later use. The surface morphology and physical properties of hybrid NPs were assessed by scanning electron microscopy (SEM) (MIRA3 LM, Czech Republic) and dynamic light scattering (DLS) (Zetasizer Nano, Malvern, UK) tools. Furthermore, HspX/EsxS encapsulation efﬁciency and *in vitro* HspX/EsxS protein release study were performed by ^125^I iodinated protein (unpublished data).

The MPLA encapsulation efﬁciency was determined by Limulus Amebocyte Lysate (LAL) kit according to the manufacturer’s protocol (Kinetic-QCL, LONZA). 

***Immunization of mice***

Mice were subcutaneously immunized with 5 mg of NP containing 10 µg HspX/EsxS fusion protein and 10 µg MPLA. 50:10 (mg) ratio of the PLGA and DDA were used for the manufacture of hybrid NP. Forty-five mice were randomly divided into 9 groups of 5 mice each and immunized subcutaneously, 200 μl/mouse, with different formulations as follows: 1) PBS (negative control), 2) 5×10^5^ CFU/mouse of BCG, 3) HspX/EsxS, 4) HspX/EsxS/MPLA, 5) PLGA/HspX/EsxS, 6) PLGA:DDA/HspX/EsxS, 7) PLGA:DDA/HspX/EsxS (booster), 8) PLGA:DDA/HspX/EsxS/MPLA and 9) PLGA:DDA/HspX/EsxS/MPLA (booster). All groups received formulations through three injections at days 0, 14 and 28. Two groups, 1 and 2, received only one time inoculation at day 0. Groups 8 and 9, in which formulations acts as booster, first received a single dose of BCG prime plus related formulation at day 0 and then formulations were administered twice with 2 weeks intervals.

***Immunoassay***

Taking blood samples and removing the spleens from the vaccinated mice were performed 3 weeks after ﬁnal vaccination. Blood samples were centrifuged to separate the sera. The concentration of anti-HspX/EsxS IgG1 and IgG2a antibodies in the sera were measured using goat anti-mouse secondary antibody, HRP conjugate through the direct ELISA method. For this purpose, ELISA 96-well plate was coated with 100 µl/well of 100 µg/ml of the purified HspX/EsxS protein diluted in 10 ml of the carbonate/bicarbonate buffer (pH 9.6) and incubated overnight at 2-8 ^°^C. The plate was washed 3 times with 0.05% Tween 20 wash buffer. Plate was blocked with 200 µl/well of blocking buffer (2% bovine serum albumin, BSA) and incubated for 1 hr at 37 ^°^C. After 5 times washing, 100 µl/well of serial dilutions of serum samples were added and incubated for 2.5 hr at 37 ^°^C. After washing, 100 µl/well of 1:500 of IgG1 and IgG2a secondary antibodies were added into related samples and again incubated for 2 hr at 37 ^°^C. After five times washing, 100 µl/well of TMB (3, 3’, 5, 5’-tetramethylbenzidine) solution was added and incubated for 15 min at room temperature. The reaction was ended by stop solution (3 N HCL) and then absorptions were read at 450 nm by microplate reader. In this study, the measured final dilution of serum samples immunized mouse with negative control (PBS) were considered as the end point titers. 

After bleeding, spleens were removed aseptically and after homogenization, extracted splenocytes were treated with lysis buffer (ammonium chloride) for the lysing of the erythrocytes. Isolated leukocyte cells were cultured in duplicate in 24-well plates in 1 ml of RPMI 1640 solution (2×10^6^ cell/well) supplemented with 10% fetal bovine serum (Gibco, UK), 1% penicillin/streptomycin (Biosera, UK) and 5 µg/ml of HspX/EsxS fusion protein or 5 µg/ml of phytohemagglutinin (PHA) (Gibco, Uk) as mitogen. After incubation for 72 hr (37 ^°^C, 5% CO_2_), culture supernatants were harvested and levels of IFN-γ, IL-4, IL-17 and TGF-β cytokines were measured by indirect ELISA method (eBioscience, USA) based on manufacturer’s guidelines.

***Statistical analysis***

All data analysis were performed by GraphPad InStat software version 3 and then expressed as mean± standard error (SEM) or mean±standard deviation SD. Tukey’s multiple comparison tests of one-way analysis of variance (ANOVA) were used to evaluate significant differences between groups. If *P*≤0.05, the results were considered as statistically significant.

## Results

***Characteristics of PLGA and PLGA:DDA hybrid NPs***

In the present study, physical characteristics of PLGA and PLGA:DDA hybrid NPs including mean diameter (Z-average, nm), surface charge (Zeta-potential, mV), polydispersity index (PDI), encapsulation efficiency (%) and yield (%) were listed in Table 1. As shown in Table 1, addition of DDA led to increase in PDI and surface charge of NPs and decreased the size, encapsulation efficiency and yield. The surface morphology and HspX/EsxS protein release profile has been shown in Figures 1 and 2. NPs were in spherical shape and with smooth surface.* In vitro* release profile of HspX/EsxS in a 15 ml release medium (PBS, pH 7.4) showed that during 1 month PLGA NPs have faster release profile than PLGA:DDA NPs.

***Determination of Th1 immune response***

Splenocytes of BALB/c mice immunized with different formulations were stimulated with HspX/EsxS and PHA and production of IFN-γ, as a Th1 marker, was assessed. As shown in Figure 3, mice subcutaneously immunized with PLGA:DDA/HspX/EsxS/MPLA (*P*<0.001) and PLGA:DDA/HspX/EsxS/MPLA (booster) (*P*<0.001) showed a significantly higher levels of IFN-γ secretion compared with BCG group. As well, these two formulations showed a significant higher levels (*P*<0.001) of IFN-γ secretion compared with the all other groups. Mice immunized with PLGA:DDA/HspX/EsxS, PLGA:DDA/HspX/EsxS (booster) and also HspX/EsxS/MPLA have higher levels of IFN-γ secretion than BCG. But, this difference was not statistically significant (*P*>0.05).

***Determination of Th2 immune response***

In all immunization groups, IL-4 levels, as a marker of Th2 immune response, was less than 5 pg/ml and showed no significant difference with BCG group (*P*>0.05) (Figure 4). However, there was statistically significant correlation between the all groups received vaccine formulations through subcutaneous injection and PBS group (*P*<0.01). In addition, by comparing the results of IL-4 and IFN-γ (IFN-γ/IL-4 ratio), in all groups, the levels of IFN-γ secretion was very higher than IL-4 concentration (data not shown). The results revealed that all formulations were able to elicit the Th1 immune responses higher than the Th2 responses.

***Determination of Th17 immune response***

As can be seen in Figure 5, in all groups, the level of IL-17, as marker of Th17, was significantly lower than BCG group (*P*<0.001). The secretion of IL-17 in all groups were higher than the PBS group (*P*<0.01).

***Determination of T-reg immune response***

TGF-β is a marker of T-reg immune response and the cytokine secretion pattern after stimulation of mice splenocytes with HspX/EsxS showed a high levels of responses, however, differences were not significantly different (*P*>0.05).

***Serum anti-HspX/EsxS IgG2a titers***

Serum anti-HspX/EsxS specific IgG2a titers (end point titers) were determined by an ELISA method. The highest IgG2a antibody titers was observed in groups subcutaneously immunized with PLGA:DDA/HspX/EsxS/MPLA and PLGA:DDA/HspX/EsxS/MPLA (booster) (*P*<0.001) as well as PLGA:DDA/HspX/EsxS and PLGA:DDA/HspX/EsxS (booster) (*P*<0.001) (Figure 7). Subcutaneous administration of these formulations induced significantly higher levels of IgG2a responses compared with the control groups (BCG and PBS) (Figure 7).

***Serum anti-HspX/EsxS IgG1 titers***

Serum anti-HspX/EsxS specific IgG1 titers (end point titers) were determined by an ELISA method. As shown in Figure 8, the highest level of IgG1 was observed in the sera of mice immunized with PLGA:DDA/HspX/EsxS/MPLA and PLGA:DDA/HspX/EsxS/MPLA (booster) as well as PLGA:DDA/HspX/EsxS and PLGA:DDA/HspX/EsxS (booster) (*P*<0.001).

## Discussion

A WHO’s global TB program called the end TB strategy was set which covers the period 2016–2030 to end the global TB epidemic, an 80% and 90% reduction in TB incidence and TB deaths, respectively (2). To achieve this goal, development of a safe, effective and high quality and cost-effective vaccine against TB is essential. The protein subunit vaccines and recently multistage subunit vaccines are among the most important TB candidate vaccines which have been evaluated in different stages of clinical trial (29). Hybrid 56 in IC31 (SSI/Intercell/Aeras) as well as ID93 in GLA-SE (IDRI) are two multistage subunit vaccines in combination with adjuvants which entered in phase I clinical trial (29). In a review study conducted by Khademi *et al*. all evaluated multistage subunit vaccines to boost immune responses against TB, alone or in combination with adjuvants, were able to: 1) strongly induce humoral and cellular immune responses, 2) reduce the bacterial load, 3) potentiate the immunogenicity of early BCG vaccine and 4) increase the protective immunity against early and latent TB infections as well as multidrug resistant *M. tuberculosis* (MDR-TB) strains (30).

Previous studies conducted by Yousefi-Avarvand* et al*. Villarreal* et al*. Yuan* et al*. Shi* et al*. Jeon* et al.* and Spratt* et al.* were examined immunogenicity profile of HspX and ESAT-6 like proteins of *M. tuberculosis* as suitable candidates for vaccination against TB infection (12, 31-35). In the current research, these two proteins which are expressed in early and latent stages were cloned and expressed as a multistage fusion protein and mice were vaccinated through subcutaneous route, three times at 2 week intervals. The results revealed that after subcutaneous injection, HspX/EsxS fusion protein alone was able to appropriately induce antigen-specific Th1, Th2, Th17 cells as well as IgG2a and IgG1 antibody responses. However, except for Th17 responses, it induced lower responses than BCG vaccine (*P*<0.001). 

For improving the immunogenicity of these multistage subunit vaccine of TB and induce stronger immune responses, effective adjuvants or appropriate delivery systems are needed (13, 14). As expected, addition of MPLA adjuvant to fusion protein, HspX/EsxS/MPLA, enhanced the IFN-γ concentration compared with HspX/EsxS protein and BCG vaccine. But, this difference was not statistically significant (*P*>0.05). Similar results were observed in our previous study when the HspX/EsxS protein subcutaneously administered along with DOTAP adjuvants (36).

Similar to MPLA responses, PLGA/HspX/EsxS formulation induced higher levels of IFN-γ cytokine as well as IgG2a and IgG1 antibody responses than HspX/EsxS antigen and BCG, however, this difference was not significant (*P*>0.05).

At the present study, PLGA and PLGA:DDA NPs were prepared and examined for their characteristics including the surface morphology, physicochemical characteristics and their immunoadjuvant potential after subcutaneous administration to BALB/c mice. A key factor to evaluate the adjuvant activity of PLGA NPs is particle size (19). Particle size and surface charge of NPs have an important role in efficient uptake by APCs and following stimulation of the immune responses necessary to increase immune responses against TB (19, 37-39). In the current research, the average particle size were 316.7 ± 12.7 and 249.7 ± 16 for PLGA and PLGA:DDA hybrid NPs, respectively. This shows that when DDA was added to PLGA matrix, particle size is reduced. Similar results have been shown by Jensen *et al.* and Kim* et al*. They showed that addition of the cationic compounds such as dioleoyltrimethylammoniumpropane (DOTAP) and polyethylenimine (PEI) to the PLGA matrix lead to significant decrease in particle size (20, 40). However, opposite results were reported by Kirby *et al* (38).

Surface charge of the modified PLGA NPs was increased when the DDA was added and changed from -33±1.7 mV to 39±1.8 mV. Compared with negatively charged PLGA, positively charged PLGA:DDA NPs shows some advantages including more stability, efficient interaction and cellular uptake by dendritic cells (DCs), better antigen adsorption, enhanced endosomal escape and more sustained antigen release (20, 25, 41). 

More stimulation of humoral (IgG1) and cell-mediated immunity (IFN-γ, IgG2a) by PLGA:DDA formulations in comparison to PLGA formulations could be attributed to the above potentials of PLGA:DDA NPs and also smaller size of PLGA:DDA NPs (38).

The present study showed that mice receiving PLGA:DDA/HspX/EsxS/MPLA formulation as well as PLGA:DDA/HspX/EsxS/MPLA (booster) generated robust antigen-specific IFN-γ and IgG2a titers as compared with all other groups (*P*<0.001). It can be concluded that the ability of the formulations containing PLGA:DDA to induce cellular immune responses is higher than PLGA formulations (*P*<0.001). As well, it is proven that IL-17 cytokine, similar to IFN-γ, has an important role in protection against TB infection. This cytokine plays a critical role in the formation of granulomas during TB infection and induce and sustain strong memory T cell responses (42). Hoft *et al.* reported that subcutaneous injection of NPs containing Ag85B along with CpG were not able to induce of IL-17 (43). Similar results were obtained in this study.

## Conclusion

PLGA:DDA NPs with optimum size, surface charge and release profile were prepared. These cationic PLGA NPs were used as a delivery system/adjuvant for encapsulation of and immunization against a novel multistage subunit vaccine (HspX/EsxS) of *M. tuberculosis*. After subcutaneous administration, both PLGA:DDA/HspX/EsxS/MPLA and PLGA:DDA/HspX/EsxS/MPLA (booster) as well as PLGA:DDA/HspX/EsxS and PLGA:DDA/HspX/EsxS (booster) are likely good subunit vaccine against TB due to induce an effective IFN-γ and IgG2a immune responses with a low increase in IL-4 and IgG1. It could be concluded that these formulations have potentials to be used as a complementary vaccine of BCG. 

## Coflicts of Interest

None to declare.

## References

[1] Bivas-Benita M, Lin MY, Bal SM, van Meijgaarden KE, Franken KL, Friggen AH (2009). Pulmonary delivery of DNA encoding Mycobacterium tuberculosis latency antigen Rv1733c associated to PLGA–PEI nanoparticles enhances T cell responses in a DNA prime/protein boost vaccination regimen in mice. Vaccine.

[2] WHO Tuberculosis. Global Tuberculosis Report 2016.

[3] Karimi SM, Sankian M, Khademi F, Tafaghodi M (2016). Chitosan (CHT) and trimethylchitosan (TMC) nanoparticles as adjuvant/delivery system for parenteral and nasal immunization against Mycobacterium tuberculosis (MTb) ESAT-6 antigen. Nanomed J.

[4] Khademi F, Derakhshan M, Sadeghi R (2016). The role of toll-like receptor gene polymorphisms in tuberculosis susceptibility: a systematic review and meta-analysis. Rev Clin Med.

[5] Khademi F, Yousefi-Avarvand A, Derakhshan M, Vaez H, Sadeghi R (2017). Middle East Mycobacterium tuberculosis Antibiotic Resistance: A Systematic Review and Meta-Analysis. Infect Epidemiol Microbiol.

[6] Niu H, Hu L, Li Q, Da Z, Wang B, Tang K (2011). Construction and evaluation of a multistage Mycobacterium tuberculosis subunit vaccine candidate Mtb10. 4–HspX. Vaccine.

[7] Ziv E, Daley CL, Blower S (2004). Potential public health impact of new tuberculosis vaccines. Emerg Infect Dis.

[8] Dey B, Jain R, Gupta UD, Katoch V, Ramanathan V, Tyagi AK (2011). A booster vaccine expressing a latency-associated antigen augments BCG induced immunity and confers enhanced protection against tuberculosis. PloS one.

[9] Brennan MJ, Clagett B, Fitzgerald H, Chen V, Williams A, Izzo AA (2012). Preclinical evidence for implementing a prime-boost vaccine strategy for tuberculosis. Vaccine.

[10] Xin Q, Niu H, Li Z, Zhang G, Hu L, Wang B (2013). Subunit vaccine consisting of multi-stage antigens has high protective efficacy against Mycobacterium tuberculosis infection in mice. PloS one.

[11] Khademi F, Yousefi-Avarvand A, Derakhshan M, Meshkat Z, Tafaghodi M, Ghazvini K (2017). Mycobacterium tuberculosis HspX/EsxS Fusion Protein: Gene Cloning, Protein Expression, and Purification in Escherichia coli. Rep Biochem Mol Biol.

[12] Yousefi-Avarvand A, Tafaghodi M, Soleimanpour S, Khademi F (2018). HspX protein as a candidate vaccine against Mycobacterium tuberculosis: an overview. Front Biol.

[13] Marongiu L, Donini M, Toffali L, Zenaro E, Dusi S (2013). ESAT-6 and HspX improve the effectiveness of BCG to induce human dendritic cells-dependent Th1 and NK cells activation. PloS one.

[14] Ottenhoff TH, Kaufmann SH (2012). Vaccines against tuberculosis: where are we and where do we need to go?. PLoS Pathog.

[15] Junqueira-Kipnis AP, Neto LMM, Kipnis A (2014). Role of fused Mycobacterium tuberculosis immunogens and adjuvants in modern tuberculosis vaccines. Front Immunol.

[16] Peek LJ, Middaugh CR, Berkland C (2008). Nanotechnology in vaccine delivery. Adv Drug Deliv Rev.

[17] Ilyinskii PO, Roy CJ, O’Neil CP, Browning EA, Pittet LA, Altreuter DH (2014). Adjuvant-carrying synthetic vaccine particles augment the immune response to encapsulated antigen and exhibit strong local immune activation without inducing systemic cytokine release. Vaccine.

[18] Kim M-G, Park JY, Shon Y, Kim G, Shim G, Oh Y-K (2014). Nanotechnology and vaccine development. Asian J Pharm Sci.

[19] Rose F, Wern JE, Ingvarsson PT, van de Weert M, Andersen P, Follmann F (2015). Engineering of a novel adjuvant based on lipid-polymer hybrid nanoparticles: A quality-by-design approach. J Control Release.

[20] Jensen DK, Jensen LB, Koocheki S, Bengtson L, Cun D, Nielsen HM (2012). Design of an inhalable dry powder formulation of DOTAP-modified PLGA nanoparticles loaded with siRNA. J Control Release.

[21] Tafaghodi M, Jaafari MR, Eskandari M, Khamesipour A (2010). Immunization against leishmaniasis by PLGA nanospheres loaded with an experimental autoclaved Leishmania major (ALM) and Quillaja saponins. Trop Biomed.

[22] Mohajer M, Khameneh B, Tafaghodi M (2014). Preparation and characterization of PLGA nanospheres loaded with inactivated influenza virus, CpG-ODN and Quillaja saponin. Iran J Basic Med Sci.

[23] Mohaghegh M, Tafaghodi M (2011). Dextran microspheres could enhance immune responses against PLGA nanospheres encapsulated with tetanus toxoid and Quillaja saponins after nasal immunization in rabbit. ‎Pharm Dev Technol.

[24] Khademi F, Derakhshan M, Yousefi-Avarvand A, Tafaghodi M (2018). Potential of polymeric particles as future vaccine delivery systems/adjuvants for parenteral and non-parenteral immunization against tuberculosis: A systematic review. Iran J Basic Med Sci.

[25] Hu Y, Ehrich M, Fuhrman K, Zhang C (2014). In vitro performance of lipid-PLGA hybrid nanoparticles as an antigen delivery system: lipid composition matters. Nanoscale Res Lett.

[26] Sayın B, Somavarapu S, Li XW, Sesardic D, Şenel S, Alpar OH (2009). TMC–MCC (N-trimethyl chitosan–mono-N-carboxymethyl chitosan) nanocomplexes for mucosal delivery of vaccines. Eur J Pharm Sci.

[27] Vyas SP, Quraishi S, Gupta S, Jaganathan K (2005). Aerosolized liposome-based delivery of amphotericin B to alveolar macrophages. Int J Pharm.

[28] Khademi F, Taheri RA, Momtazi-Borojeni AA, Farnoosh G, Johnston TP, Sahebkar A (2018). Potential of Cationic Liposomes as Adjuvants/Delivery Systems for Tuberculosis Subunit Vaccines. Rev Physiol Biochem Pharmacol.

[29] Kaufmann SH (2013). Tuberculosis vaccines: time to think about the next generation. Semin Immunol.

[30] Khademi F, Derakhshan M, Yousefi-Avarvand A, Tafaghodi M, Soleimanpour M (2018). Multi-stage subunit vaccines against Mycobacterium tuberculosis: An alternative to the BCG vaccine or a BCG-prime boost?. Expert Rev Vaccines.

[31] Villarreal DO, Walters J, Laddy DJ, Yan J, Weiner DB (2014). Multivalent TB vaccines targeting the esx gene family generate potent and broad cell-mediated immune responses superior to BCG. Hum Vaccin Immunother.

[32] Yuan W, Dong N, Zhang L, Liu J, Lin S, Xiang Z (2012). Immunogenicity and protective efficacy of a tuberculosis DNA vaccine expressing a fusion protein of Ag85B-Esat6-HspX in mice. Vaccine.

[33] Shi C, Chen L, Chen Z, Zhang Y, Zhou Z, Lu J (2010). Enhanced protection against tuberculosis by vaccination with recombinant BCG overexpressing HspX protein. Vaccine.

[34] Jeon B-Y, Kim S-C, Eum S-Y, Cho S-N (2011). The immunity and protective effects of antigen 85A and heat-shock protein X against progressive tuberculosis. Microb Infec.

[35] Spratt JM, Britton WJ, Triccas JA (2010). In vivo persistence and protective efficacy of the Bacille Calmette Guerin vaccine overexpressing the HspX latency antigen. Bioeng bugs.

[36] Khademi F, Sahebkar A, Fasihi-Ramandi M, Taheri RA (2018). Induction of strong immune response against a multicomponent antigen of Mycobacterium tuberculosis in BALB/c mice using PLGA and DOTAP adjuvant. Apmis.

[37] Richter WF, Jacobsen B (2014). Subcutaneous absorption of biotherapeutics: knowns and unknowns. ‎Drug Metab Dispos.

[38] Kirby DJ, Rosenkrands I, Agger EM, Andersen P, Coombes AG, Perrie Y (2008). PLGA microspheres for the delivery of a novel subunit TB vaccine. J Drug Target.

[39] Khademi F, Taheri RA, Avarvand AY, Vaez H, Momtazi-Borojeni AA, Soleimanpour S (2018). Are chitosan natural polymers suitable as adjuvant/delivery system for anti-tuberculosis vaccines?. Microb Pathog.

[40] Kim I-S, Lee S-K, Park Y-M, Lee Y-B, Shin S-C, Lee KC (2005). Physicochemical characterization of poly (L-lactic acid) and poly (D, L-lactide-co-glycolide) nanoparticles with polyethylenimine as gene delivery carrier. Int J Pharm.

[41] He C, Hu Y, Yin L, Tang C, Yin C (2010). Effects of particle size and surface charge on cellular uptake and biodistribution of polymeric nanoparticles. Biomaterials.

[42] Verwaerde C, Debrie A-S, Dombu C, Legrand D, Raze D, Lecher S (2014). HBHA vaccination may require both Th1 and Th17 immune responses to protect mice against tuberculosis. Vaccine.

[43] Hoft DF, Worku S, Kampmann B, Whalen CC, Ellner JJ, Hirsch CS (2002). Investigation of the relationships between immune-mediated inhibition of mycobacterial growth and other potential surrogate markers of protective Mycobacterium tuberculosis immunity. J Infect Dis.

